# Supplementary material for: A highly attenuated Vesiculovax vaccine rapidly protects nonhuman primates against lethal Marburg virus challenge
Source: PLoS Negl Trop Dis. 2022 May 27;16(5):e0010433. doi: 10.1371/journal.pntd.0010433 (PMC9182267; doi:10.1371/journal.pntd.0010433)
Supplement: S1 Table — Macaques were immunized with a vector control (n = 1) or rVSV-N4CT1-MARV-GP vaccine at -7 DPI (n = 5). *Day after MARV challenge is in parentheses up to the 28 DPI study endpoint. †Fever is defined as a temperature greater than 2.5 °F above baseline, at least 1.5 °F above baseline and ≥ 103.5 °F, or 1.1 °F above baseline and ≥ 104°F. Leukopenia, thrombocytopenia, and lymphopenia are defined by a > 40% drop in numbers of leukocytes, platelets, and lymphocytes, respectively. Leukocytosis, monocytosis, and granulocytosis are defined as a ≥ two-fold increase in leukocytes, monocytes, and granulocytes, respectively. Crosses indicate increases in liver enzymes (ALT, AST, ALP, GGT) or renal function test values (BUN, CRE): 2- to 3-fold increase, +; >3- up to 5-fold increase, ++; >5-fold increase, +++. Abbreviations: M, male; F, female; kg, kilogram; PFU, plaque-forming units; MARV, Marburg virus; BUN, blood urea nitrogen; CRE, creatinine; ALT, alanine aminotransferase; AST, aspartate aminotransferase; ALP, alkaline phosphatase; GGT, gamma-glutamyltransferase; CRP, c-reactive protein; DPI, days post infection. (DOCX) [file pntd.0010433.s005.docx]

| Animal ID  (sex) weight | Group  (Day of Vaccination) | RT-qPCR Titer (LOG_10_ copies/ml)* | Viremia Titer (LOG_10_ PFU/ml)* | Clinical Signs*† | Final Outcome |
| --- | --- | --- | --- | --- | --- |
| Control 1  (M) 2.86 kg | -7 | 7.25 (3), 11.88 (6), 11.71 (8) | 5.08 (3), 8.72 (6), 8.43 (8) | Anorexia (7,8), petechial rash (8), dyspnea (8), lymphopenia (3), lymphocytosis + (8), monocytosis + (6) +++ (8), neutropenia (8), neutrophilia + (0,3,6), eosinopenia (0,3), eosinophilia +++ (8), basopenia (3), basophilia + (8), BUN ++ (8), CRE +++ (8), ALT +++ (6,8), AST +++ (6,8), ALP ++ (6,8), GGT +++ (6,8), CRP increase (6,8) | Euthanized 8 DPI |
| Survivor 1  (M) 3.36 kg | -7 | N.D. | N.D. | Monocytopenia (0,14), neutrophilia ++ (0,14), eosinopenia (21), basopenia (21) | Survived |
| Survivor 2  (F) 3.88 kg | -7 | N.D. | N.D. | Monocytopenia (14) | Survived |
| Survivor 3  (M) 7.60 kg | -7 | N.D. | N.D. | Leukopenia (0,6,10,14,21,28), lymphocytosis + (10), monocytopenia (14,28), neutropenia (0,3,6,10,14,21,28), eosinopenia (0,6,10,14,21), basopenia (0,3,6,10,14,21,28) | Survived |
| Survivor 4  (F) 3.94 kg | -7 | N.D. | N.D. | Anorexia (5), neutropenia (28), eosinopenia (6), basopenia (6), ALT + (0,3) ++ (10,21,28) +++ (14), CRP increase (6) | Survived |
| Survivor 5  (F) 3.20 kg | -7 | N.D. | N.D. | Neutropenia (0,10), eosinopenia (0,21), eosinophilia + (28), basopenia (0,21) | Survived |

**S1 Table. Clinical findings in MARV-exposed cynomolgus macaques immunized with Vesiculovax vaccine 7 days prior to challenge.**

Macaques were immunized with a vector control (n=1) or rVSV-N4CT1-MARV-GP vaccine at -7 DPI (n=5). *Day after MARV challenge is in parentheses up to the 28 DPI study endpoint. †Fever is defined as a temperature greater than 2.5 °F above baseline, at least 1.5 °F above baseline and ≥ 103.5 °F, or 1.1 °F above baseline and ≥ 104°F. Leukopenia, thrombocytopenia, and lymphopenia are defined by a > 40% drop in numbers of leukocytes, platelets, and lymphocytes, respectively. Leukocytosis, monocytosis, and granulocytosis are defined as a ≥ two-fold increase in leukocytes, monocytes, and granulocytes, respectively. Crosses indicate increases in liver enzymes (ALT, AST, ALP, GGT) or renal function test values (BUN, CRE): 2- to 3-fold increase, +; >3- up to 5-fold increase, ++; >5-fold increase, +++. Abbreviations: M, male; F, female; kg, kilogram; PFU, plaque-forming units; MARV, Marburg virus; BUN, blood urea nitrogen; CRE, creatinine; ALT, alanine aminotransferase; AST, aspartate aminotransferase; ALP, alkaline phosphatase; GGT, gamma-glutamyltransferase; CRP, c-reactive protein; DPI, days post infection.
