# Supplementary material for: A highly attenuated Vesiculovax vaccine rapidly protects nonhuman primates against lethal Marburg virus challenge
Source: PLoS Negl Trop Dis. 2022 May 27;16(5):e0010433. doi: 10.1371/journal.pntd.0010433 (PMC9182267; doi:10.1371/journal.pntd.0010433)
Supplement: S2 Table — Macaques were immunized with a vector control (n = 1) or rVSV-N4CT1-MARV-GP vaccine at -5 DPI (n = 5). *Day after MARV challenge is in parentheses up to the 28 DPI study endpoint. †Fever is defined as a temperature greater than 2.5 °F above baseline, at least 1.5 °F above baseline and ≥ 103.5 °F, or 1.1 °F above baseline and ≥ 104°F. Leukopenia, thrombocytopenia, and lymphopenia are defined by a > 40% drop in numbers of leukocytes, platelets, and lymphocytes, respectively. Leukocytosis, monocytosis, and granulocytosis are defined as a ≥ two-fold increase in leukocytes, monocytes, and granulocytes, respectively. Crosses indicate increases in liver enzymes (ALT, AST, ALP, GGT) or renal function test values (BUN, CRE): 2- to 3-fold increase, +; >3- up to 5-fold increase, ++; >5-fold increase, +++. Abbreviations: M, male; F, female; kg, kilogram; PFU, plaque-forming units; MARV, Marburg virus; BUN, blood urea nitrogen; CRE, creatinine; ALT, alanine aminotransferase; AST, aspartate aminotransferase; ALP, alkaline phosphatase; GGT, gamma-glutamyltransferase; CRP, c-reactive protein; DPI, days post infection. (DOCX) [file pntd.0010433.s006.docx]

| Animal ID (sex) weight | Group  (Day of Vaccination) | RT-qPCR Titer (LOG_10_ copies/ml)* | Viremia Titer (LOG_10_ PFU/ml)* | Clinical Signs*† | Final Outcome |
| --- | --- | --- | --- | --- | --- |
| Control 2  (F) 3.44 kg | -5 | 10.64 (6), 11.45 (8) | 7.83 (6), 7.68 (8) | Fever (6), anorexia (8), severe depression (8), petechial rash (7,8), dyspnea (8), bleeding at venipuncture site (8), leukocytosis ++ (8), lymphocytosis + (8), monocytosis + (3) +++ (8), neutropenia (0), neutrophilia + (6) +++ (8), eosinopenia (0), eosinophilia + (8), basophilia ++ (8), BUN ++ (8), CRE + (0,3,6) +++ (8), ALT +++ (6,8), AST +++ (6,8), ALP + (6) ++ (8), GGT +++ (8), CRP increase (6,8) | Euthanized 8 DPI |
| Survivor 6  (M) 3.50 kg | -5 | N.D. | N.D. | Monocytosis + (0,14), neutrophilia + (0), eosinopenia (14,28), basopenia (28) | Survived |
| Survivor 7  (F) 3.20 kg | -5 | N.D. | N.D. | Monocytosis +++ (6), neutrophilia + (6), eosinopenia (0), basopenia (0) | Survived |
| Survivor 8  (M) 3.66 kg | -5 | N.D. | N.D. | Monocytosis + (3,6) ++ (0), eosinopenia (6,10,21,28), eosinophilia + (3), basopenia (6,10,21) | Survived |
| Fatal 1  (F) 3.44 kg | -5 | 7.56 (6), 11.88 (9) | 4.02 (6), 8.26 (9) | Fever (3,6), anorexia (6,7,8,9), mild depression (3,6), severe depression (9), uncoordinated movement (8,9), petechial rash (9), thrombocytopenia (6), lymphopenia (6), monocytopenia (0), neutrophilia + (3,9), eosinopenia (3,6), basopenia (6), BUN ++ (9), CRE +++ (9), ALT +++ (9), AST +++ (9), ALP ++ (6) +++ (9), GGT +++ (9), amylase +++ (9), CRP increase (3,6,9) | Euthanized 9 DPI |
| Survivor 9  (M) 3.32 kg | -5 | N.D. | N.D. | Leukocytosis + (3), monocytosis + (3,6,14), neutropenia (28), neutrophilia + (3), eosinopenia (6,21,28) | Survived |

**S2 Table. Clinical findings in MARV-exposed cynomolgus macaques immunized with Vesiculovax vaccine 5 days prior to challenge.**

Macaques were immunized with a vector control (n=1) or rVSV-N4CT1-MARV-GP vaccine at -5 DPI (n=5). *Day after MARV challenge is in parentheses up to the 28 DPI study endpoint. †Fever is defined as a temperature greater than 2.5 °F above baseline, at least 1.5 °F above baseline and ≥ 103.5 °F, or 1.1 °F above baseline and ≥ 104°F. Leukopenia, thrombocytopenia, and lymphopenia are defined by a > 40% drop in numbers of leukocytes, platelets, and lymphocytes, respectively. Leukocytosis, monocytosis, and granulocytosis are defined as a ≥ two-fold increase in leukocytes, monocytes, and granulocytes, respectively. Crosses indicate increases in liver enzymes (ALT, AST, ALP, GGT) or renal function test values (BUN, CRE): 2- to 3-fold increase, +; >3- up to 5-fold increase, ++; >5-fold increase, +++. Abbreviations: M, male; F, female; kg, kilogram; PFU, plaque-forming units; MARV, Marburg virus; BUN, blood urea nitrogen; CRE, creatinine; ALT, alanine aminotransferase; AST, aspartate aminotransferase; ALP, alkaline phosphatase; GGT, gamma-glutamyltransferase; CRP, c-reactive protein; DPI, days post infection.
