# Supplementary material for: A highly attenuated Vesiculovax vaccine rapidly protects nonhuman primates against lethal Marburg virus challenge
Source: PLoS Negl Trop Dis. 2022 May 27;16(5):e0010433. doi: 10.1371/journal.pntd.0010433 (PMC9182267; doi:10.1371/journal.pntd.0010433)
Supplement: S3 Table — Macaques were immunized with a vector control (n = 1) or rVSV-N4CT1-MARV-GP vaccine at -3DPI (n = 5). *Day after MARV challenge is in parentheses up to the 28 DPI study endpoint. †Fever is defined as a temperature greater than 2.5 °F above baseline, at least 1.5 °F above baseline and ≥ 103.5 °F, or 1.1 °F above baseline and ≥ 104°F. Leukopenia, thrombocytopenia, and lymphopenia are defined by a > 40% drop in numbers of leukocytes, platelets, and lymphocytes, respectively. Leukocytosis, monocytosis, and granulocytosis are defined as a ≥ two-fold increase in leukocytes, monocytes, and granulocytes, respectively. Crosses indicate increases in liver enzymes (ALT, AST, ALP, GGT) or renal function test values (BUN, CRE): 2- to 3-fold increase, +; >3- up to 5-fold increase, ++; >5-fold increase, +++. Abbreviations: M, male; F, female; kg, kilogram; PFU, plaque-forming units; MARV, Marburg virus; BUN, blood urea nitrogen; CRE, creatinine; ALT, alanine aminotransferase; AST, aspartate aminotransferase; ALP, alkaline phosphatase; GGT, gamma-glutamyltransferase; CRP, c-reactive protein; DPI, days post infection. (DOCX) [file pntd.0010433.s007.docx]

| Animal ID (sex) weight | Group  (Day of Vaccination) | RT-qPCR Titer (LOG_10_ copies/ml)* | Viremia Titer (LOG_10_ PFU/ml)* | Clinical Signs*† | Final Outcome |
| --- | --- | --- | --- | --- | --- |
| Control 3  (F) 2.96 kg | -3 | 11.70 (6), 11.09 (9) | 2.00 (3), 8.33 (6), 7.08 (9) | Anorexia (8), mild depression (8,9), petechial rash (6,7,8,9), diarrhea (8,9), leukocytosis ++ (9), thrombocytopenia (9), lymphopenia (6), lymphocytosis ++ (9), monocytosis + (3) +++ (9), neutrophilia + (9), eosinopenia (0,3,6), eosinophilia +++ (9), basopenia (0,6), basophilia ++ (9), ALT +++ (6,9), AST +++ (6,9), ALP + (9) ++ (6), GGT ++ (6,9), CRP increase (6) | Euthanized 9 DPI |
| Fatal 2  (M) 6.80 kg | -3 | 9.52 (6) | 1.40 (3), 5.98 (6) | Fever (6), anorexia (6), petechial rash (6), bleeding nares (6), thrombocytopenia (6), CRE ++ (6), ALT +++ (6), AST +++ (6), ALP ++ (6), CRP increase (6) | Euthanized 6 DPI |
| Fatal 3  (M) 3.00 kg | -3 | 5.58 (3), 10.18 (6) | 2.97 (3), 6.34 (6) | Fever (3,6), anorexia (5,6), moderate depression (6), petechial rash (6), rectal bleeding (6), bleeding at venipuncture sites (6), thrombocytopenia (6), lymphopenia (3), monocytopenia (0,3), neutrophilia + (3), eosinopenia (0,3,6), basopenia (0,3,6), BUN + (6), CRE + (6), ALT +++ (6), AST +++ (6), GGT ++ (6), CRP increase (3,6) | Euthanized 6 DPI |
| Fatal 4  (F) 2.84 kg | -3 | 6.00 (3), 11.36 (6), 9.23 (7) | 3.78 (3), 8.13 (6), 8.16 (7) | Anorexia (5,6,7), severe depression (7), mild petechial rash (6,7), rectal bleeding (7), leukocytosis + (7), lymphopenia (0,3), lymphocytosis + (7), monocytosis + (7), neutrophilia + (3,7), eosinopenia (0,3,6), basopenia (3,6), basophilia + (7), ALT +++ (6), AST +++ (6), ALP ++ (6), GGT ++ (6), CRP increase (3,6) | Euthanized 7 DPI |
| Survivor 10  (M) 6.70 kg | -3 | 6.76 (6), 7.90 (10), 5.48 (14) | 2.89 (3), 3.34 (6), 4.26 (10) | Anorexia (6,7,8,9,10), petechial rash (9,10,11,12), leukocytosis + (3,28) +++ (14), lymphopenia (6), lymphocytosis + (21) ++ (14), monocytosis + (3,21) ++ (14,28), neutrophilia + (6,10,28) ++ (3) +++ (14), eosinopenia (6), eosinophilia + (14) ++ (28), basopenia (6), basophilia + (3,28) ++ (14), BUN ++ (10), CRE + (10), ALT + (21) +++ (10,14), AST + (6) ++ (14) +++ (10), ALP + (6) ++ (28) +++ (10,14,21), GGT + (28) ++ 21) +++ (10,14), CRP increase (6,10) | Survived |
| Fatal 5  (F) 3.24 kg | -3 | 5.05 (3), 10.22 (6), 8.62 (7) | 2.44 (3), 8.49 (6), 7.32 (7) | Anorexia (6,7), petechial rash (6,7), dyspnea (7), thrombocytopenia (6,7), lymphopenia (3,6), neutrophilia + (3,7), eosinophilia + (3) ++ (7), BUN + (6) +++ (7), CRE ++ (6), ALT +++ (6,7), AST +++ (6,7), ALP +++ (6,7), GGT +++ (6,7), amylase + (7), CRP increase (3,6,7) | Euthanized 7 DPI |

**S3 Table. Clinical findings in MARV-exposed cynomolgus macaques immunized with Vesiculovax vaccine 3 days prior to challenge.**

Macaques were immunized with a vector control (n=1) or rVSV-N4CT1-MARV-GP vaccine at -3DPI (n=5). *Day after MARV challenge is in parentheses up to the 28 DPI study endpoint. †Fever is defined as a temperature greater than 2.5 °F above baseline, at least 1.5 °F above baseline and ≥ 103.5 °F, or 1.1 °F above baseline and ≥ 104°F. Leukopenia, thrombocytopenia, and lymphopenia are defined by a > 40% drop in numbers of leukocytes, platelets, and lymphocytes, respectively. Leukocytosis, monocytosis, and granulocytosis are defined as a ≥ two-fold increase in leukocytes, monocytes, and granulocytes, respectively. Crosses indicate increases in liver enzymes (ALT, AST, ALP, GGT) or renal function test values (BUN, CRE): 2- to 3-fold increase, +; >3- up to 5-fold increase, ++; >5-fold increase, +++. Abbreviations: M, male; F, female; kg, kilogram; PFU, plaque-forming units; MARV, Marburg virus; BUN, blood urea nitrogen; CRE, creatinine; ALT, alanine aminotransferase; AST, aspartate aminotransferase; ALP, alkaline phosphatase; GGT, gamma-glutamyltransferase; CRP, c-reactive protein; DPI, days post infection.
